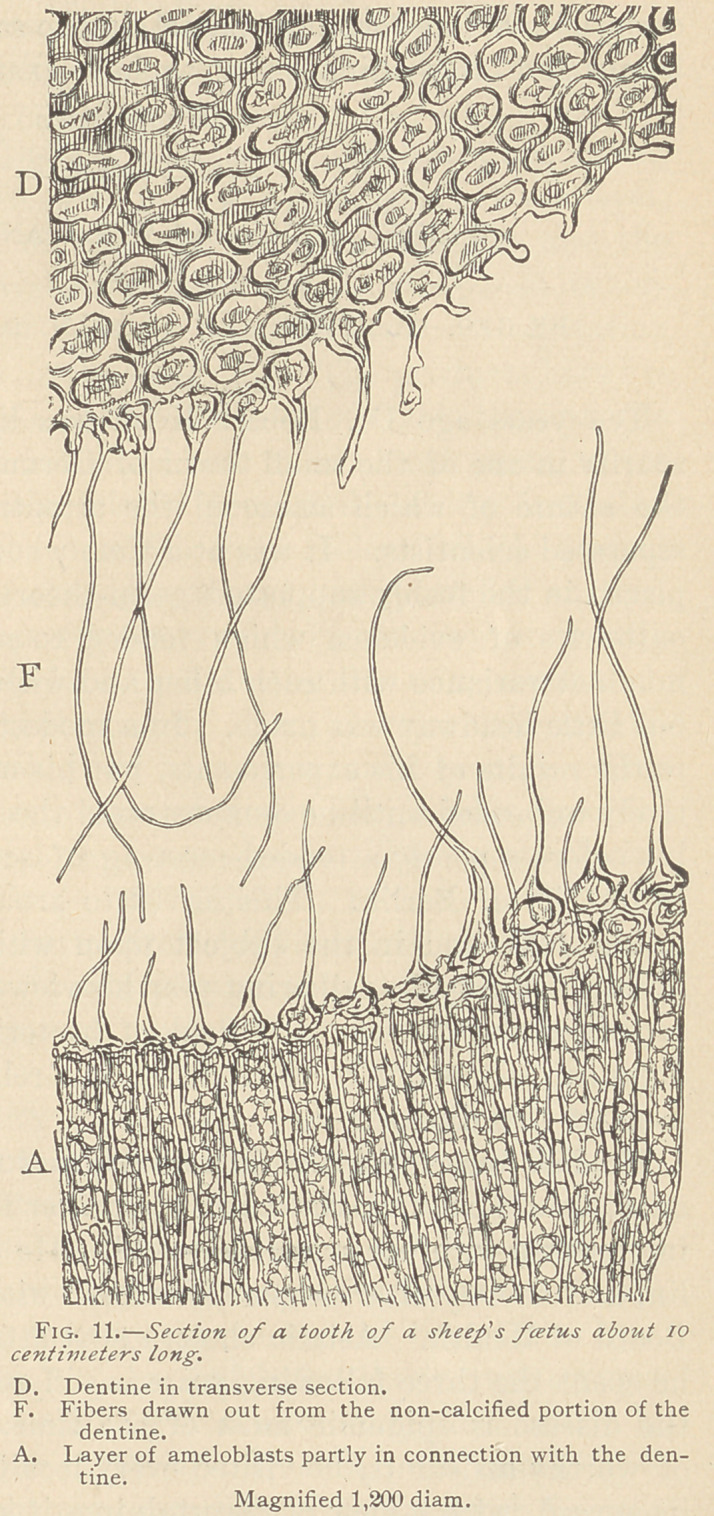# Contributions to the History of Development of the Teeth

**Published:** 1887-06

**Authors:** Carl Heitzmann, C. F. W. Bödecker


### Independent Practitioner.

VOL. VIII. JUNE, 1887. NO. 6.

NOTE.—No paper published or to be published in another journal will be accepted for this department. All papers must be in the hands of the Editor before the first day of the month preceding that in which they are expected to appear. Extra copies will be furnished to each contributor of an accepted original article, and reprints, in pamphlet form, may be had at the cost of the paper, press-work and binding, if ordered when the manuscript is forwarded. The Editor and Publishers are not responsible for the opinions expressed by contributors. The journal is issued promptly, on the first day of each month.

### Original Communications.

### CONTRIBUTIONS TO THE HISTORY OF DEVELOPMENT OF THE TEETH.

BY CARL HEITZMANN, M. D., AND C. F. W. BÖDECKER, D. D. S., M. D. S.

CONTINUED FROM PAGE 235.

The formation of enamel commences about the sixth month of fœtal life, at a period when the dentine, which begins to form about the fifth month, has assumed a certain thickness. About the seventh month, we observe at the summit of the papilla a comparatively broad cap, the dentine, and above this a somewhat narrower layer of enamel. (Fig. 7.)

Investigations of the development of enamel are rendered difficult by the fact that the enamel organ is almost invariably found to be detached from the enamel and the papilla. This perplexity may be obviated, at least to a certain extent, by filling the cavity between the enamel organ and the enamel with celloidine. The cavity or space is evidently the result of shrinkage of the delicate myxomatous tissue in the enamel organ. A peculiar asymmetry

FIG. 7. *First formed dentine and enamel of human fœtus, seven months.*

E. Enamel cap.

D. Calcified dentinal cap.

DD. Non-calcified dentine.

O. Row of odontoblasts.

P. Papilla, with blood-vessels.

M. Stellate reticulum of enamel organ.

IE. Inner epithelium.

EE. Clusters of the remains of the outer epithelium.

C. Fibrous connective tissue.

Magnified 50 diam.

is often met with, one side being very broad, and the other very narrow. (See Fig. 7.) Peculiar indentations, also, are often seen in the course of the inner epithelium. Whether or not all of this is artificial, and due to shrinkage, we are unable to state, but it is certain that as soon as the enamel begins to appear, there are marked differences in the structure of the inner epithelium. At the bottom of the enamel organ, corresponding to the future neck of the tooth, the inner epithelium is found to be transformed into medullary tissue, and the edge between the inner and outer epithelium is likewise filled with medullary tissue. Higher up, along the border of the papilla, where there is as yet no enamel, we find formations resembling the original epithelia, but which in this condition are termed ameloblasts. The intermediate layer, in connection with the ameloblasts in this stage, is always markedly developed. Still higher up, where ready-formed enamel is present, the ameloblasts are much less regular, and we observe that they again split up into medullary corpuscles. From this we draw the conclusion that the original inner epithelium is transformed into medullary corpuscles, which give rise to the ameloblasts, and that the latter, before being transformed into enamel, once more break up into medullary tissue.

In a specimen prepared from a human fœtus of five months, there is scarcely a trace of the inner epithelium left. There we see nothing of the tissue except a succession of small glistening medullary corpuscles, which, by their arrangement in rows, remind us of their origin from previous epithelia. This tissue remains plainly visible, even in the sixth and seventh month of fœtal life especially, as mentioned above, at the fold corresponding to the edge of the enamel organ in the region of the future neck of the tooth. Higher up, the medullary corpuscles, which previously were scattered, once more assume a line-like arrangement, and gradually assume the shape of narrow elongated corpuscles, not always distinctly nucleated, and these elongated bodies, somewhat resembling the original columnar epithelia, are termed ameloblasts. (Fig. 8.)

One of the striking features in this process of transformation is the presence of a layer forming the outermost portion of the enamel organ toward the papilla. (Fig. 8, P.) This layer, with low powers of the microscope, appears to be made up of finely granular protoplasm with interspersed granular nuclei. Above this, marks of division appear in the protoplasmic layer, and the marks correspond to the boundary lines of the rows of the glistening medul-

Fig. 8. *Segment near the fold of the enamel organ, corresponding to the neck of the future tooth of a human fœtus of the seventh month.*

PP. Papilla.

S. Stellate reticulum in an early stage of development.

M. Medullary corpuscles in a nearly uniform distribution, becoming elongated higher up, and arranged in rows.

A. Ameloblasts at an early stage of formation, composed of medullary corpuscles.

P. Protoplasmic bodics which produce the outermost portion of the enamel organ.

Magnified 1,200 diam.

lary corpuscles, the forerunners of the future ameloblasts. There is a transitional stage, as referred to in Fig. 8, in which the inner portions of the ameloblasts are made up of several glistening medullary corpuscles, whereas the outermost portion is finely granular, or made up of a delicate reticulum. The ameloblasts in the seventh month of fœtal life are distinctly marked formations, and are present on such places only where enamel has not yet been formed. They join in length from the region of the neck to the summit of the crown. They are composed of finely granular protoplasm, with one or several nuclei, or sometimes without any distinct nucleus. Their general form is columnar, slightly broadened toward the papilla. Often, however, they exhibit parallel contours, or a wedge-shape, between two neighboring funnel-shaped ameloblasts. (See Fig. 6, A.) All ameloblasts are interconnected by delicate conical threads traversing the light interstices between them. Their layer is easily distinguished from the stellate reticulum by the intermediate layer, which is composed of spindles and fibers. From their bases delicate short offshoots often emanate (Tomes' processes), which, however, do not exhibit any regular arrangement. Where the layer of ameloblasts is detached from the surface of the papilla, similar short processes emanate from the surface of the latter, and it is obvious that all these fine offshoots serve for an interconnection between the ameloblasts, and the medullary corpuscles of the papilla.

Still nearer the summit of the crown the ameloblasts once more lose their character, and once more break up into medullary corpuscles, more or less retaining their row-like arrangement. We observe that the medullary corpuscles, which lie nearest to the already formed dentine, are finely granular, whereas the rows some distance above are coarsely granular or homogeneous. These finely granular medullary corpuscles are at last infiltrated with lime-salts, and thus is produced the enamel proper. (Fig. 9.)

The first trace of enamel, as is well known, appears about the sixth month of intra-uterine life, at a period when a certain amount of dentine has already been formed. The outer surface of the dentine exhibits bay-like excavations, in which we often observe a flat layer of a finely granular protoplasm, analogous to that found in the teeth of adults. Above this layer we see prismatic pieces of calcified basis-substance irregularly distributed, since the enamel prisms, as a rule, do not reach the surface of the dentine, but are replaced by a homogeneous (Tomes' granular) layer. Yet above this the enamel prisms are easily recognizable, and appear to be composed of more or less regular square pieces. In the latter instance we can see a deposition of lime-salts along the borders of the prisms, whereas their central portions often exhibit one large nucleus, or several coarse granules. The interstices between the rows of these square pieces frequently exhibit delicate fibrillæ, the enamel fibers, which are in connection with broader protoplasmic tracts,

FIG. 9.—*First formed enamel of a human fœtus of six months.*

D. Dentine bounded toward the enamel by bay-like excavations.

E. Enamel composed of prismatic pieces.

A. Rows of medullary corpuscles sprung from previous ameloblasts.

Magnified 1000 Diam.

lying in the boundary between the enamel and dentine. These enamel fibers send branches through the transverse interstices of the square pieces. Everywhere delicate offshoots are seen, indicating that the living matter of the previous medullary corpuscles is preserved, even after their infiltration with lime-salts.

In the eighth and ninth months of fœtal life, both the enamel and the dentine form solid caps, corresponding to the summit of the crown of the tooth, and are super-imposed upon each other. In decalcified specimens of these tissues, when they have been stained with carmine or chloride of gold, we observe a striking analogy in the structure of both the enamel and the dentine. With a magnifying power of 500 diameters, it is difficult to differentiate between dentine and enamel. The fibers in the canaliculi of the dentine closely resemble those of the interstices between the enamel rods. Even the highest powers of the microscope exhibit a close resemblance of both tissues, more especially after the lime-salts have been completely removed by means of reagents.

The sum of the results of our researches concerning the development of the enamel in the human subject may be condensed as follows:

1. In the third month of intra-uterine life there begins to appear between the inner and outer epithelium the so-called stellate reticulum.

2. The stellate reticulum is a myxomatous arisen from a medullary tissue, into which portions of both the inner and the outer epithelium are transformed.

3. The myxomatous tissue is the enamel organ proper, and although arisen from epithelia, is a variety of connective tissue.

4. Both the nucleated reticulum and the basis-substance of the meshes are crowded with living matter, which, in turn, after being reduced to medullary tissue, is transformed into enamel.

5. The external epithelium, by multiplication of its elements, produces buds directed outward.

6. The buds of the external epithelium break up into medullary tissue, accompanied by free vascularization (formation of blood-vessels and blood corpuscles) of the adjacent fibrous connective tissue.

7. The connective tissue sprung from the external epithelium is probably the material from which the enamel originates after the exhaustion of the central myxomatous tissue.

8. About the fifth month of fœtal life, the inner epithelium breaks completely up into medullary corpuscles, which are characterized by their small size and a nearly homogeneous appearance.

9. By a rearrangement of these medullary corpuscles, slender columnar bodies arise, the so-called ameloblasts, which precede the formation of the enamel.

10. The ameloblasts are again broken up into rows of medullary corpuscles, which by direct calcification produce the enamel prisms, and thus the subdivision into smaller segments takes place.

Among the different animals of which we have examined the process of the formation of the enamel, we may mention the following :

Kittens at the time of birth show all stages of the formation of teeth, and especially the breaking up of the inner epithelium into

FIG. 10.—*Ameloblasts of a pig's fœtus about 10 centimeters long, before the formation of enamel.*

M. Myxomatous enamel organ.

A. Rows of ameloblasts of a markedly reticular structure.

G. Granular layer between the dentine and the ameloblasts.

Magnified 1,200 Diam.

medullary, and afterwards into myxomatous tissue, which is easily traceable. All the details, however, correspond to those of human beings.

In a newly born pup the formation of enamel was found, in all essential points, identical with that in man.

The fœtus of a pig forms an excellent object for the study of the formation of ameloblasts from medullary corpuscles. (Fig. 10.)

The ameloblasts are arranged with great regularity, being alternately wedge-shaped in opposite directions. Their reticulum is very wide, and the horizontal threads traversing the interstices quite plain. From their bases arise a varying number of offshoots, directed toward a peculiar tissue which occupies the space between the ameloblasts and the already formed dentine. This tissue appears granular with low powers of the microscope. High powers, however, plainly reveal a reticular structure, traversed by delicate fibrillæ in connection with the reticulum. The presence of nuclei in this tissue indicates its origin from medullary corpuscles.

The fœtus of a lamb about ten centimeters long shows a large layer of ameloblasts. (Fig. 11.)

In places where the ameloblasts lie close to the dentine, no fibrillæ are seen, but where the layer of ameloblasts is detached, a large number of fibrillæ appear, evidently belonging to the dentine. This is less calcified and takes up a deep carmine stain, contrary to the fully calcified central portion of the dentine, which usually remains unstained. Here a layer

D

F

A

FIG. 11.—*Section of a tooth of a sheep's fœtus about 10 centimeters long.*

D. Dentine in transverse section.

F. Fibers drawn out from the non-calcified portion of the dentine.

A. Layer of ameloblasts partly in connection with the dentine.

Magnified 1,200 diam.

is wedged in between the dentine and the ameloblasts, which is peculiar to the sheep. The ameloblasts break up into medullary corpuscles before forming enamel, in the same manner as in human beings and other animals that we have examined.

(TO BE CONTINUED.)

## Figures and Tables

**Fig. 7. f1:**
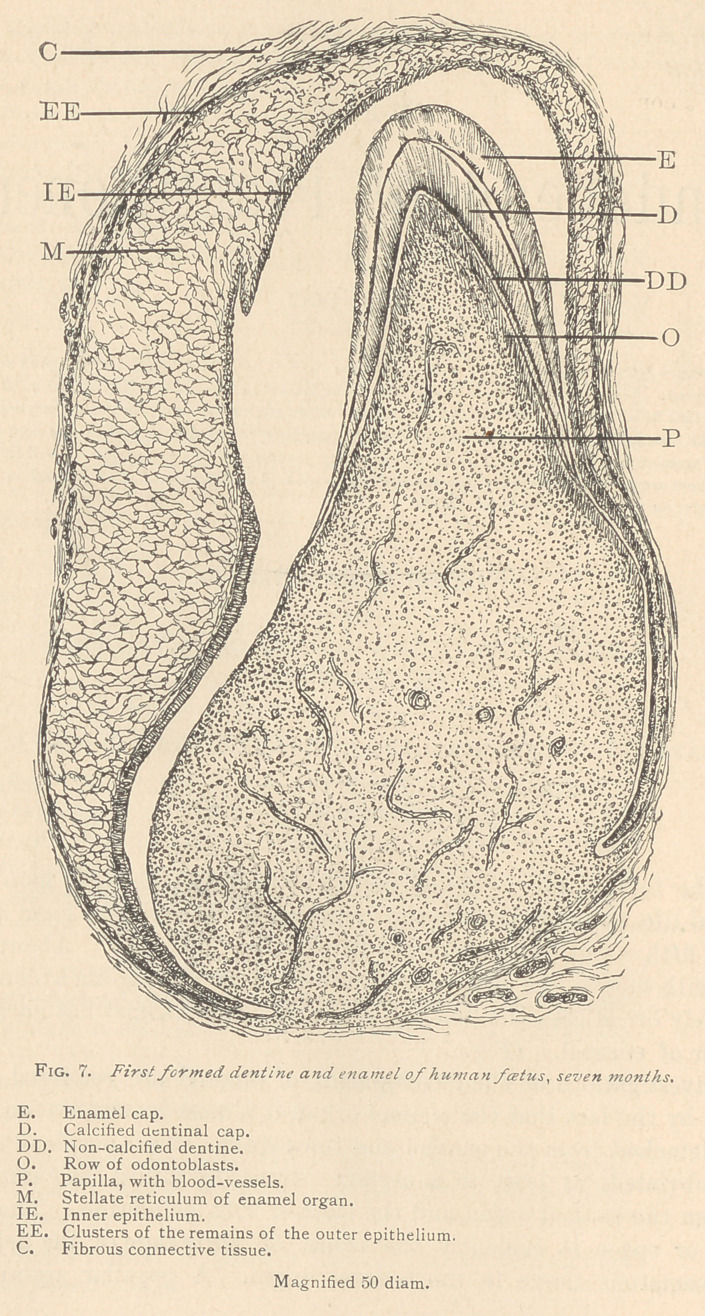


**Fig. 8. f2:**
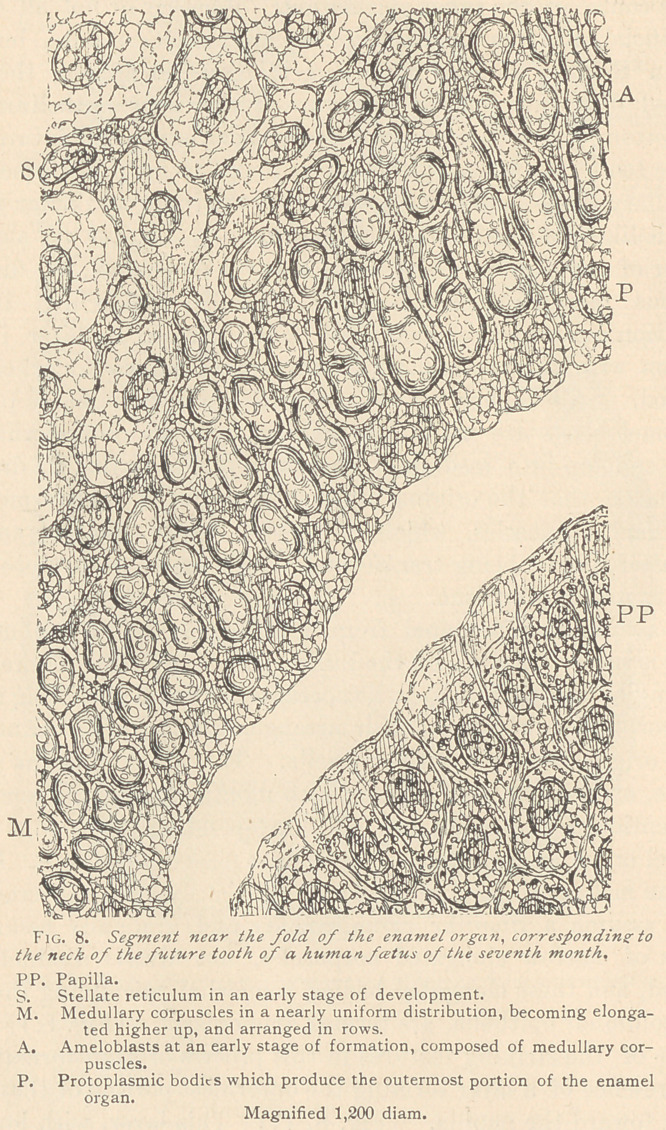


**Fig. 9. f3:**
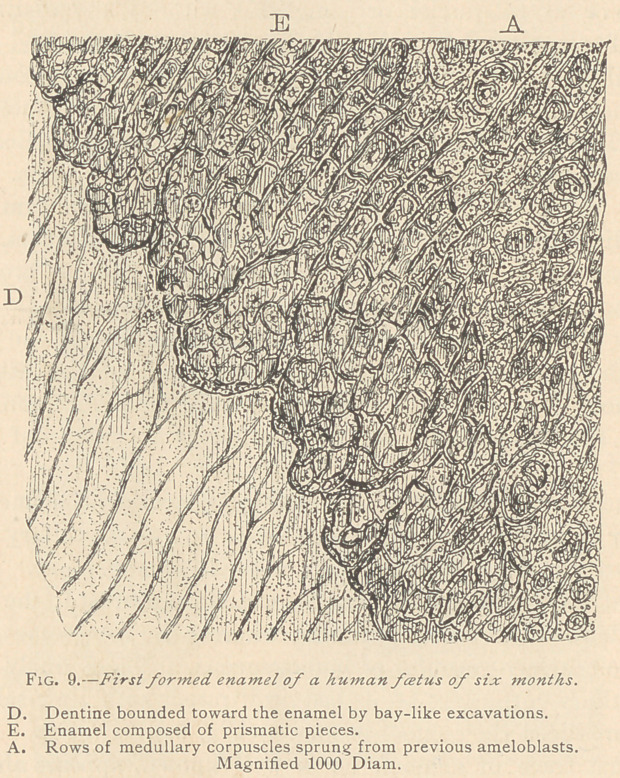


**Fig. 10. f4:**
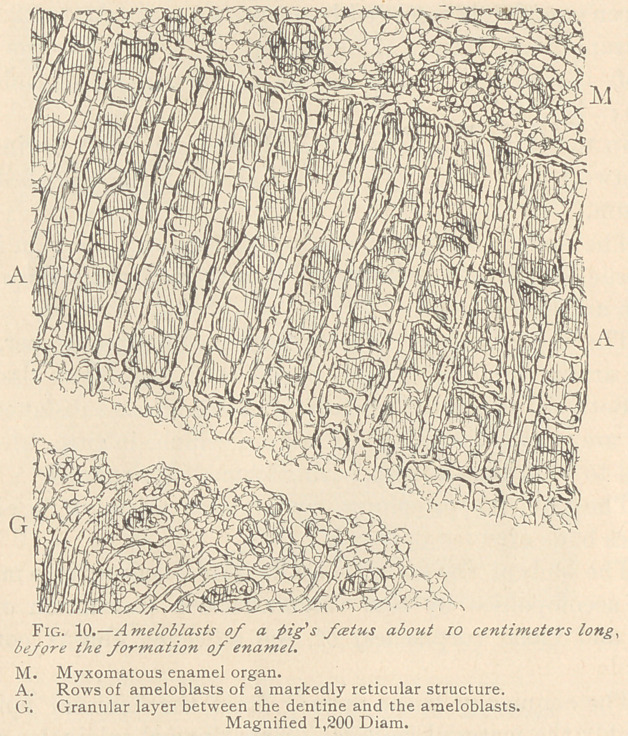


**Fig. 11. f5:**